# Full Mouth Rehabilitation With All-on-Six Concept With Computer-Aided Design/Computer-Aided Manufacturing (CAD/CAM) Milled Bar Retained Zirconia Prosthesis

**DOI:** 10.7759/cureus.67465

**Published:** 2024-08-22

**Authors:** Arushi Beri, Sweta G Pisulkar, Akansha Bansod, Sabita Rauniyar, Ritul Jain, Shruti Deshmukh

**Affiliations:** 1 Prosthodontics, Crown, and Bridge, Sharad Pawar Dental College and Hospital, Datta Meghe Institute of Higher Education and Research, Wardha, IND; 2 Orthodontics and Dentofacial Orthopedics, Kalinga Institute of Dental Science, Bhubaneswar, IND

**Keywords:** all-on-six concept, zirconia prosthesis, milled bar, cad/cam, full mouth rehabilitation

## Abstract

Full-mouth rehabilitation, also known as full-mouth reconstruction or restoration, is the comprehensive process of restoring and rehabilitating the function, aesthetics, and health of all teeth in the upper and lower jaws. This involves a multidisciplinary approach, addressing issues such as occlusion (bite), tooth wear, tooth loss, and periodontal (gum) health to achieve optimal dental function and appearance. This treatment provides a stable and reliable solution for dental problems associated with the complete or partial loss of dental elements. This case report discusses the management of a 72-year-old male patient who came with a chief complaint of decayed, missing, and mobility of his teeth. His medical history included drug-regulated diabetes and chronic periodontitis, which remained a reason behind his deteriorating dental health. The treatment plan encompassed a comprehensive approach, starting with the full-mouth extraction and progressing to the placement of six implants in both the mandibular and maxillary arches for complete restoration. After allowing eight weeks for healing, the impressions for the implants were recorded with the polyvinyl siloxane material. The case demonstrates the application of the all-on-six concept; whereby full arch rehabilitation is possible with the strategic placement of six implants. By employing the latest in advanced computer-aided design/computer-aided manufacturing (CAD/CAM), a zirconia prosthesis that was retained on a milled bar was fabricated-yielding a very high degree of accuracy and excellent aesthetics in the restoration. Restorations with zirconium were done, as it is excellent in aesthetics and functioning. This article considers the importance of precision planning, correct implant placement, and advances in dental science and technology. Most importantly, the conclusion that can be drawn is that the end outcome of total rehabilitation with all-on-six should not only be an affirmation that difficult cases can be managed very well but also the patient's quality of life is improved by using the latest dental procedures.

## Introduction

Historically, the primary options for rehabilitating an edentulous dental arch included sub-periosteal implant-retained prostheses, either removable or fixed, and conventional complete denture therapy. Subperiosteal implants, although extensively utilized in the past, presented significant problems with implant mobility and had questionable survival rates, typically only lasting between five and seven years [1-4]. Due to this, complete dentures were commonly recommended as the treatment of choice for an individual with a completely edentulous arch. Recently, a new technique, known as an all-on-six implant treatment, has evolved to replace all missing teeth in an arch [5-7]. It seems that success with this requires that the cases be selected cautiously. Thus, a surgical technique must be evaluated through radiographic and clinical findings along with careful prosthetic planning before execution. This case report details some of the surgical and prosthetic techniques of implant-supported prosthetic rehabilitation in cases with totally edentulous arches [8-10].

The following is a case report presenting a full arch rehabilitation with six endosteal implants following the standard delayed loading protocol for the rehabilitation in both the maxillary and the mandibular arches. The patient had implant-supported bar retained zirconia restorations done, utilizing advanced computer-aided design/computer-aided manufacturing (CAD/CAM) technology. This approach shows the importance of thorough planning and utilization of modern dental technologies in achieving successful full-arch rehabilitation.

## Case presentation

A 72-year-old male patient presented to the outpatient dental department with missing teeth and all the remaining teeth showing mobility (Grade III mobile 18, 32,42 and 37; Grade II mobility with 24, 25, 44, 45, 46) and had a poor prognosis along with supra eruption. His medical history revealed diabetes mellitus, which had been diagnosed eight years previously, and he was taking medication for it. The patient was dissatisfied with the aesthetics but did not want a removable prosthesis, so he was taken up for a detailed evaluation for a fixed prosthesis. For that reason, the planning of full-mouth extractions followed by immediate implant placement and bar-supported fixed prostheses for both jaws. In our patient, special attention was paid to the assessment of the smile line and transition zone during the planning phase to meet the aesthetic needs. The smile line, which refers to the curve of the upper teeth that follows the lower lip when smiling, was carefully evaluated for symmetry, shape, and position of the teeth relative to the lips and gums. This involved ensuring that the teeth were aligned symmetrically with the midline of the face, checking the position of the upper central incisors to ensure they followed a natural curve along the lower lip, and evaluating the amount of gum tissue visible when the patient smiles to avoid excessive gingival display. Additionally, the dynamics of the lips were considered to ensure that the teeth would appear natural during different facial expressions. In the transition zone, which is the area where the prosthesis meets the natural tissues, the contour of the gums was analyzed to create a seamless blend. The thickness of the gingival tissue was also assessed to predict how it would adapt to the implant-supported prosthesis, and attention was given to color matching between the prosthetic teeth and the surrounding gums. Functional aspects, such as bite alignment and the stability of the prosthesis, were also considered to ensure that the treatment would not only provide aesthetic satisfaction but also meet the patient's functional needs. By addressing these details, we were able to plan a treatment that resulted in a natural and pleasing smile.

Radiographic investigations

**Figure 1:**
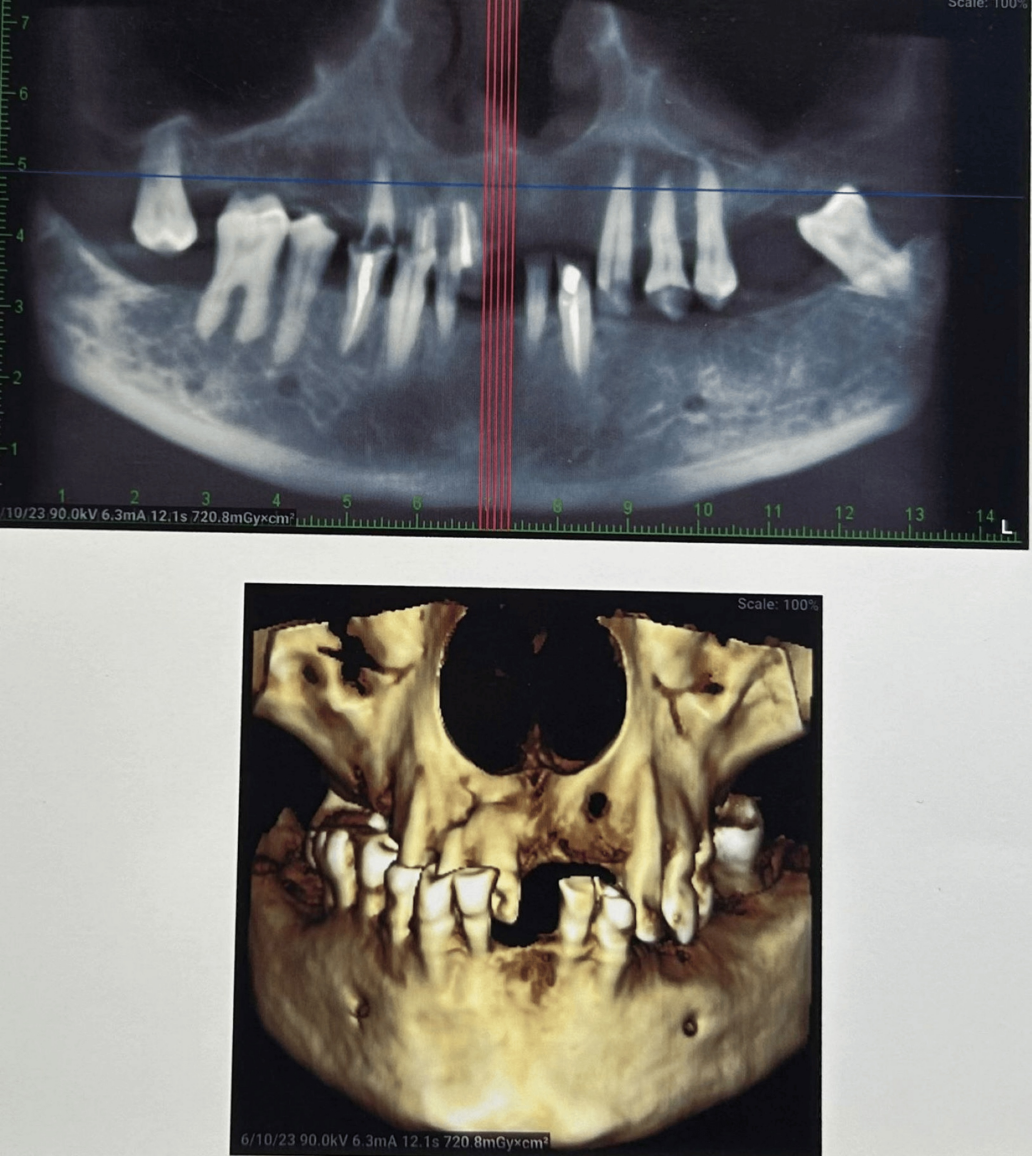
Pre-treatment radiographic evaluation

This patient's dental structures were evaluated using cone beam computed tomography (CBCT). Figure 1 depicts the radiographic evaluation for both arches, giving more detailed information regarding the periodontal status of the remaining teeth.

Surgical phase

**Figure 2:**
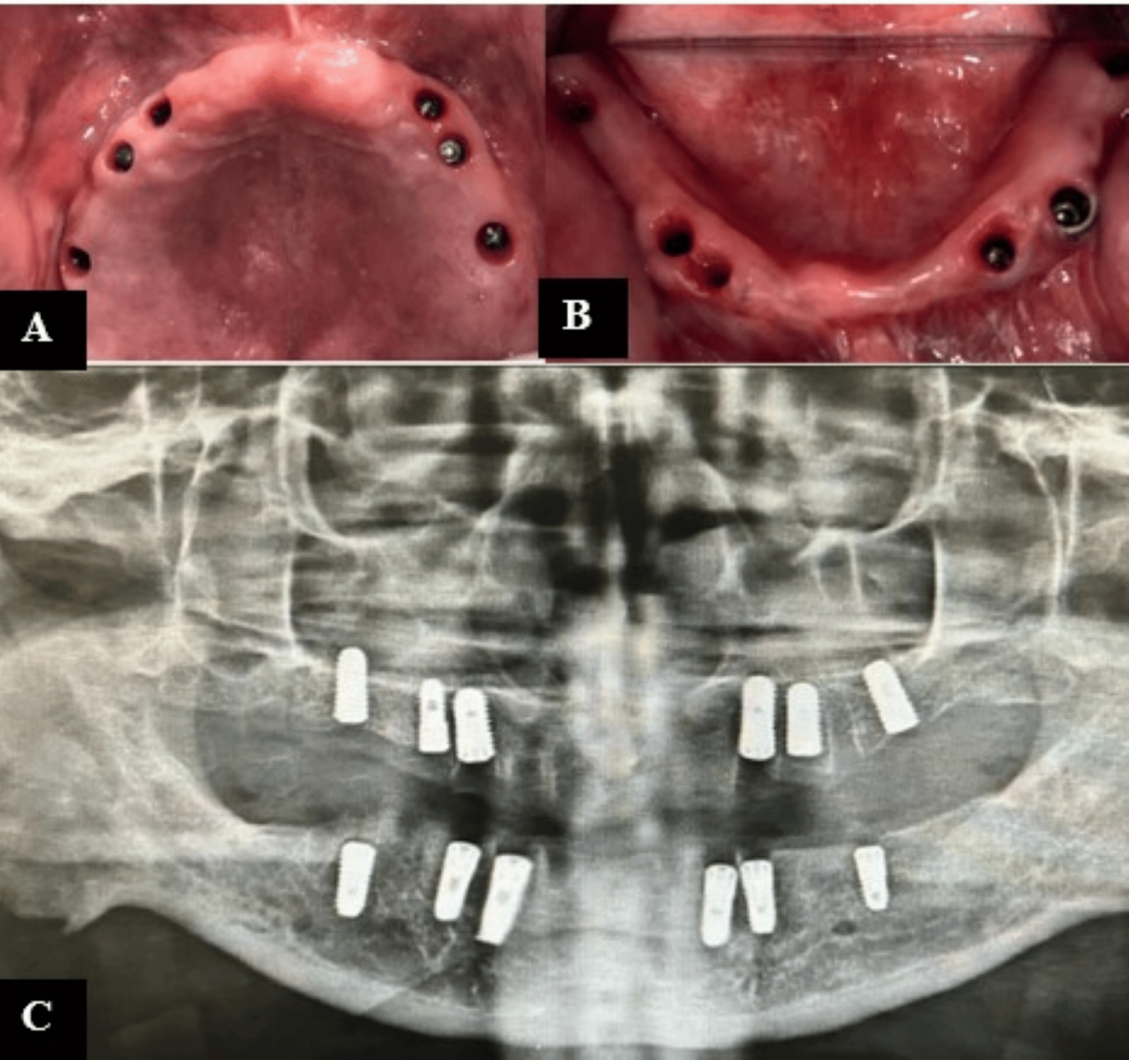
(A) Maxillary arch (six implants); (B) mandibular arch (six implants); (C) post-operative radiograph

The patient underwent a thorough preoperative evaluation, which included assessing the type of bone encountered during tooth extraction (D2 and D3 bone was present) and the proximity of the maxillary sinus. The quality and density of the alveolar bone were critical in determining the feasibility and placement of dental implants. Additionally, the proximity of the maxillary sinus played a significant role in the treatment planning as the remaining bone height was 8 mm in 16 regions so indirect sinus lift was planned, particularly for the placement of implants in the maxillary arch. Careful consideration was given to avoid sinus perforation, and if necessary, sinus augmentation procedures were planned to ensure adequate bone support for the implants. the local anesthesia was infiltrated using lignocaine and 1:100,000 adrenaline. All remaining teeth were extracted, and twelve implants were placed with primary stability recorded at 15-20 N/cm by a torque force, therefore delayed loading was planned for the patient. Cover screws were placed, and implants were submerged for three months (Figures 2A-2C). After three months of healing second stage surgery were done and the cover screw was removed healing abutments were placed for 15 days for the mucosa to heal and proper gingival collar to form.

Prosthetic phase

**Figure 3:**
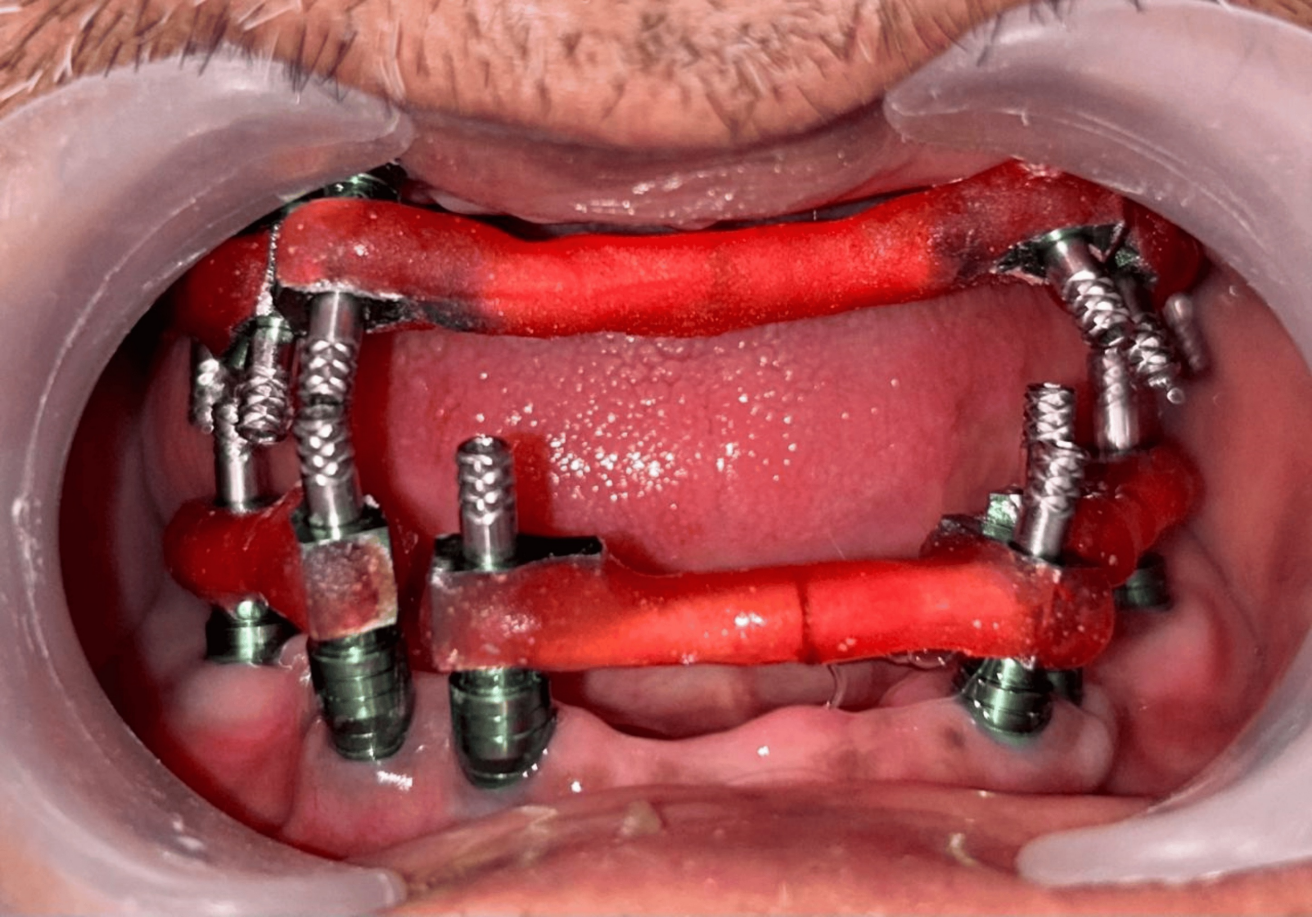
Impression copings splinted with denta floss and pattern resin

The healing abutments were removed from the implants and the open tray impression copings were selected and fitted. These impression copings were splinted together using dental floss and pattern resin intraorally for added stability and precision of the prosthesis (Figure 3).

**Figure 4:**
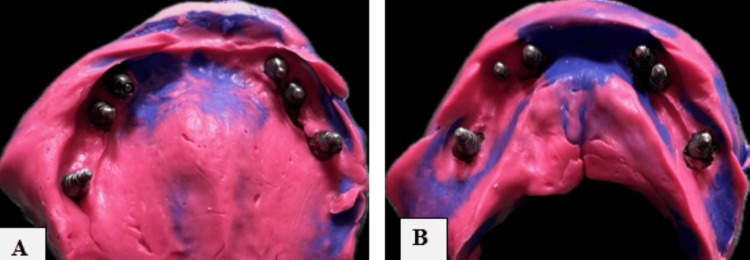
(A) Maxillary and (B) mandibular open tray impressions

The custom-made impression tray was carefully seated in the mouth, the copings were allowed to fall into the window created in the tray and then to let the impression material stabilize these copings. Final impressions were made in addition to silicone with variable viscosities (light body and putty). Once the setting time had elapsed, the impression copings were removed along with the impression, and a cast was created using a die stone. The copings were unscrewed through a window in the tray. Figures 4A, 4B depict the maxillary and mandibular impression.

**Figure 5:**
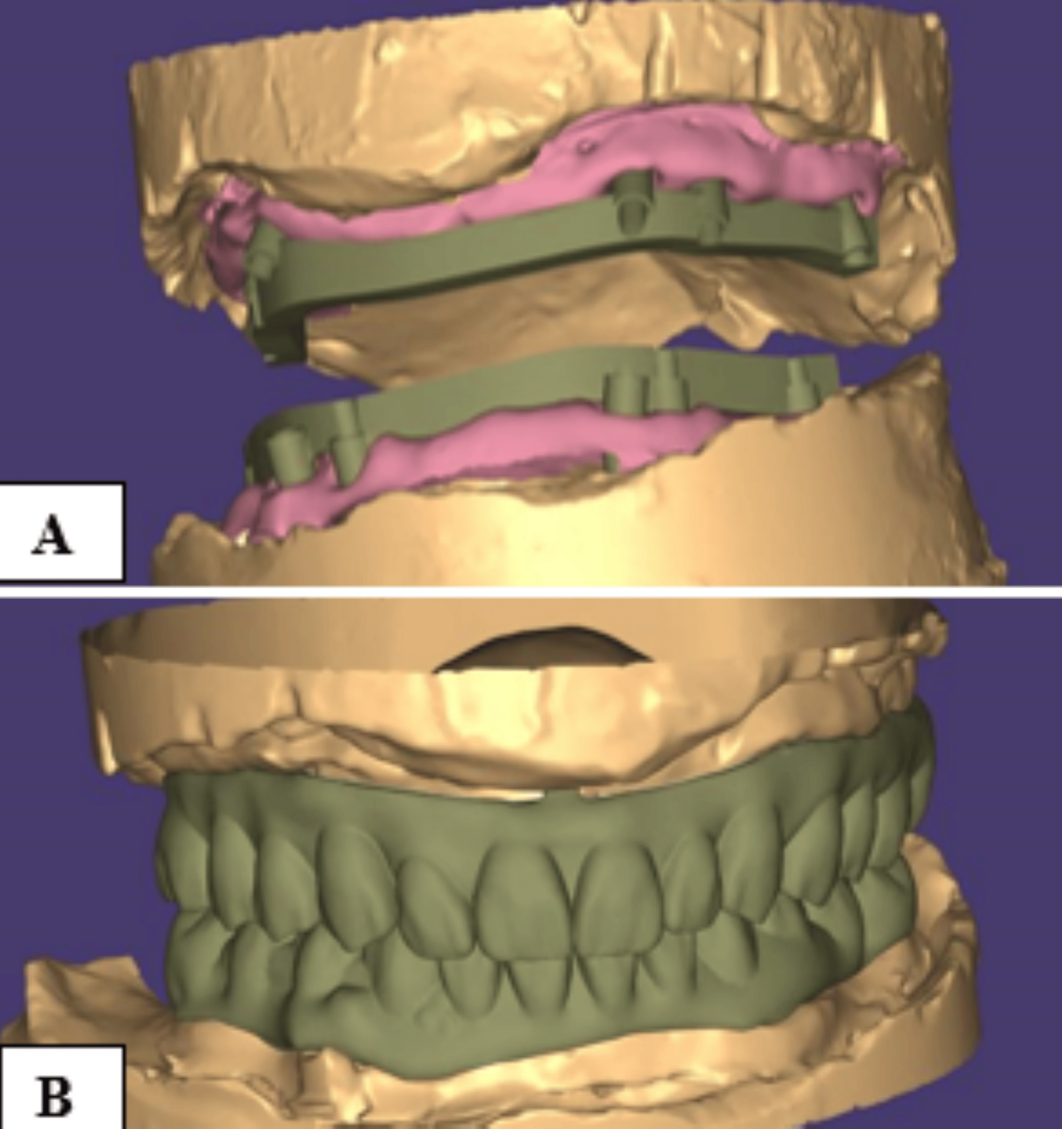
Computer-assisted designing using Exocad software - (A) bar and (B) prosthesis at occlusion

Following this, the jaw relation was recorded using wax occlusal rims and a divider, and the occlusal vertical dimension was verified. A face-bow record and a centric relation record were obtained. The mounted cast was digitized with a lab scanner and the design was done using Exocad software (Darmstadt, Germany) (Figures 5A, 5B).

Trials were done and occlusal adjustments were made. The final restorations were made of zirconia ceramic, designed in conjunction with aesthetics and function. This involved ensuring evenly distributed centric contacts and establishing anterior guidance for eccentric contacts across multiple teeth. The reduction of shear forces was achieved by maintaining shallow tooth contact angles during the disclusion of posterior teeth. Centric relation, as outlined by Dawson, was carefully considered, and the occlusal vertical dimension was adjusted to provide appropriate incisal guidance and tooth form. These occlusal adjustments ensured that the bite force applied to the prosthesis, implants, and bone was primarily compressive.

Bisque trial

**Figure 6:**
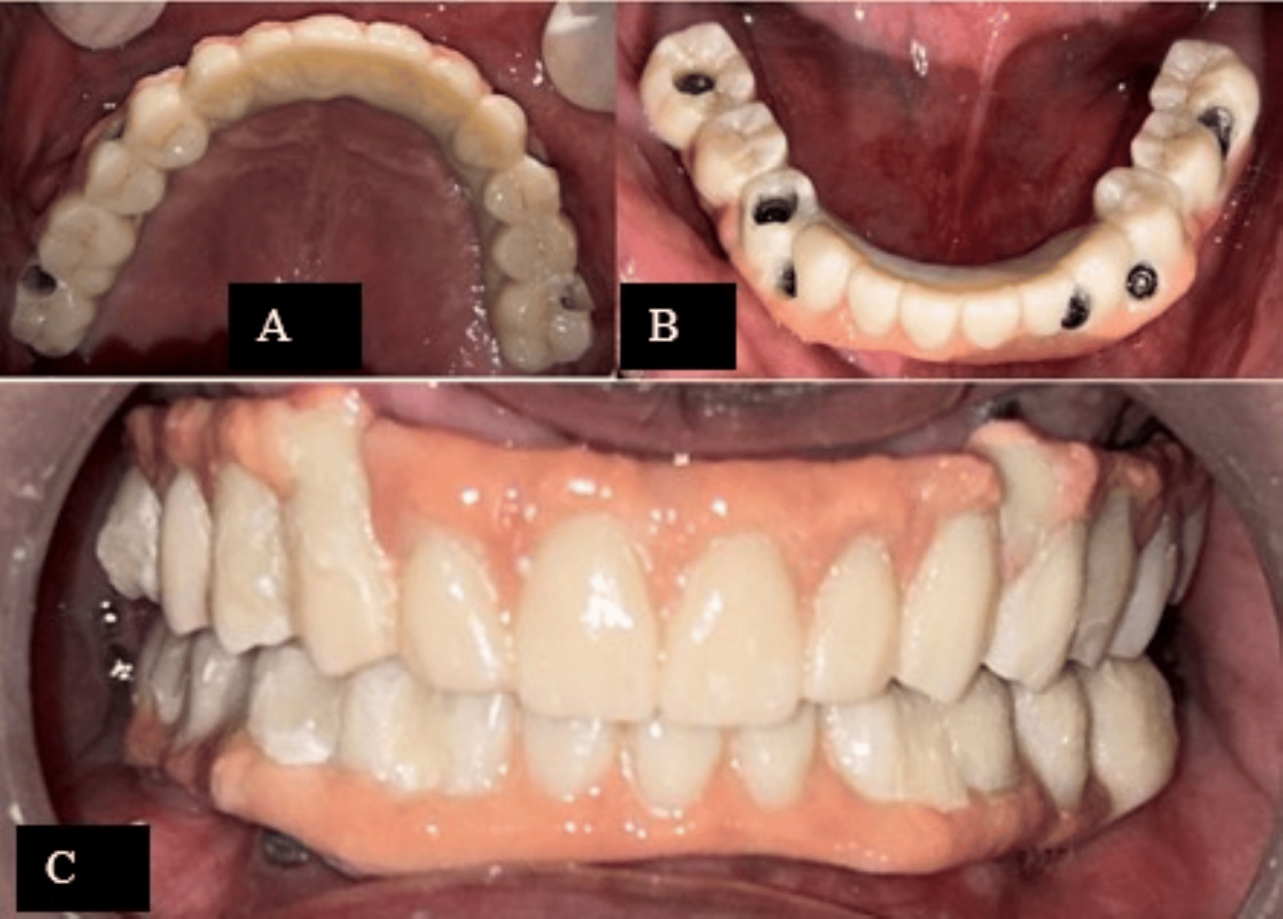
CAD/CAM milled bar retained zirconia prostheses - (A) maxillary arch; (B) mandibular arch; (C) at occlusion CAD/CAM - computer-aided design/computer-aided manufacturing

**Figure 7:**
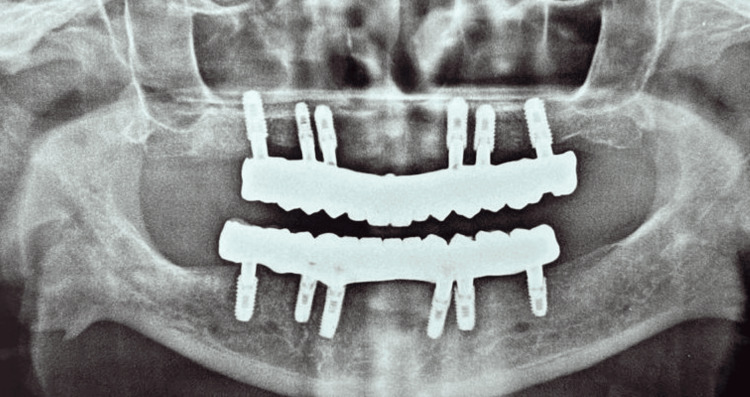
Post-operative radiograph

**Figure 8:**
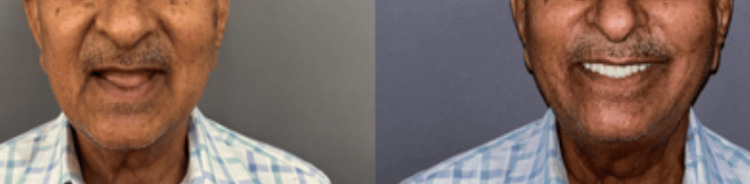
Comparison of pre- and post-operative photograph

After milling, a bisque trial was performed (Figures 6A-6C), and final adjustments were checked before screwing the prosthesis into place and covering the screws with composite material. Figure 7 shows radiographic images of the patient after placement of the prostheses and Figure 8 shows a comparison of pre- and post-operative photograph.

## Discussion

By introducing the use of four to six endosseous implants in the anterior region of the edentulous arch, in the maxilla, and mandible supporting a full-arch fixed prosthesis, the work by Brånemark et al. laid down fundamental principles for today's dental implantology [11]. Over 10 years, their study achieved a success rate of 78.3% to 80.3% for the maxilla and 88.4% to 93.2% for the mandible [12]. Our case study focuses on the rehabilitation of partial edentulism in the jaw using the all-on-six dental implant concept. Such stability and function resulted from the technique, which was promising in the possibility of restoring the normal chewing and speech abilities of the patients. One of the major benefits of the all-on-six concept is that it makes bone augmentation procedures avoidable, thus reducing morbidity, surgical time, and subsequently, recovery time [13]. Also, the implants are important in stabilization, and a fixed prosthesis can be provided immediately to establish immediate functional and aesthetic benefits. In addition, fixed prostheses individually tailored with concern to the smile line and facial structure of the patient result in improved long-term aesthetics.

This case report is a detailed elucidation of the all-on-six treatment concept. Other reports, however, have compared literature between the two concepts for the all-on-four and all-on-six concepts, and even though the all-on-six method can cost more while the treatment time can potentially take longer, it is the better approach by using more implants and a better equal distribution of stress [14]. The fact that the all-on-six concept abounds with cantilevers means that many of the biomechanical prosthetic problems have been obviated. Research by Hassan et al. has supported this. The research findings also served to support the fact that dental implant success lies within oral hygiene, therefore, having lower plaque levels in all-on-six groups when compared to all-on-four groups. That spelled out the fact that meticulous prosthesis planning and maintenance should be done for impeccable results [15].

Data by Park et al. discredit this idea of age as a limiting factor against implant success, showing that older patients can have successful implant placements with quite favorable long-term outcomes. In our patient, detailing of the smile line and transition zone was paid special attention during planning to fulfill the aesthetic needs of the patient. Zirconia was used due to its better features, while due care in occlusal adjustment ensured a satisfactory bilateral occlusion [16]. While the all-on-six is an improvement compared to traditional procedures in all areas of dental implantology regarding stability, function, and esthetics, the long-term success still lies in proper patient selection and meticulous prosthetic planning [17-21].

## Conclusions

Full-mouth dental implant rehabilitation offers an effective solution for patients who are either completely edentulous or have teeth with poor prognosis. This approach integrates principles of stability, function, and aesthetic satisfaction, and is widely regarded as a major advancement in modern dental implantology. By using six dental implants, this treatment provides strong support for a fixed prosthesis, leading to improved aesthetic outcomes and enhanced quality of life for patients. The case study demonstrates the key advantages of the all-on-six implant technique, including the reduced need for additional bone augmentation, which simplifies the surgery and accelerates healing. Consequently, this method delivers immediate enhancements in appearance and function, positively impacting the patient's self-esteem and overall well-being. The all-on-six concept represents a significant breakthrough in comprehensive dental rehabilitation, offering a holistic approach that is both functional and aesthetically pleasing. When combined with best practices in implantology and thorough patient assessment, it holds great potential for treating patients with complete and partial edentulism.
